# Cross-sectional and longitudinal functional network alterations associated with subthreshold depressive symptoms in healthy older adults

**DOI:** 10.3389/fnagi.2026.1742371

**Published:** 2026-03-11

**Authors:** Pascal Grumbach, Julia Christl, Camilla Mendl-Heinisch, Natalia Wege, Christiane Jockwitz, Eva Meisenzahl, Svenja Caspers

**Affiliations:** 1 Department of Psychiatry and Psychotherapy, Medical Faculty, Heinrich-Heine-University, Düsseldorf, Germany; 2 Institute of Neurosciences and Medicine, Brain & Behaviour (INM-7), Research Centre Jülich, Jülich, Germany; 3 Department of Psychiatry and Psychotherapy, LVR-Klinikum Essen, University of Duisburg-Essen, Medical Faculty, Essen, Germany; 4 Institute for Anatomy I, Medical Faculty and University Hospital Düsseldorf, Heinrich Heine University Düsseldorf, Düsseldorf, Germany; 5 Institute of Neurosciences and Medicine (INM-1), Research Centre Jülich, Jülich, Germany

**Keywords:** early detection, functional connectivity, late-life depression, subthreshold depressive symptoms, ventral attention network

## Abstract

**Objective:**

Subthreshold depressive symptoms (SDS) in older adults as a prodromal state of late-life depression (LLD) increase with age and are associated with elevated risk for cardio- and cerebrovascular diseases. LLD has been linked to functional brain network disruptions, including the ventral attention (VAN) and default mode network (DMN). Thus, identifying alterations in functional connectivity (FC) linked to SDS may be critical for the early detection and treatment of individuals at high-risk for LLD.

**Methods:**

A total of 243 healthy older subjects (55–84 years; M_Age_ = 67.0 ± 6.5) without a history of depression and current antidepressant intake from the 1000BRAINS sample with two timepoints of assessments were included in this study (time interval = 3.7 ± 0.7 years). SDS were measured using the Beck Depression Inventory II (BDI-II < 20) and linked to resting-state functional magnetic resonance imaging derived FC within and between seven large scale functional brain networks.

**Results:**

Both cross-sectional and longitudinal analyses revealed that SDS were associated with decreased intra- and inter-network FC. After Bonferroni correction for multiple comparisons, reduced FC within the VAN at baseline significantly predicted a longitudinal increase in depressive symptoms. This association was primarily driven by the somatic symptom domain of the BDI-II. *Post-hoc* analyses highlighted the particular involvement of right-hemispheric VAN regions.

**Conclusion:**

Our findings support the hypothesis that even minimal to mild depressive symptoms in older adults are linked to disrupted functional network architecture. Specifically, reduced FC within the VAN may serve as an early neural marker for the emergence of depressive symptoms and vulnerability for the progression into clinically manifest LLD. Thus, offering potential for early detection and targeted intervention in subjects at high risk.

## Introduction

Late-life depression (LLD) represents a prevalent and serious public health concern in aging populations, with a lifetime prevalence of approximately 13.3% (Abdoli et al., 2022). When considering depression as a dimensional diagnosis, the prevalence increases to 19.5% in older people, although there is significant heterogeneity in these estimates (Volkert et al., 2013). Thus, dimensional LLD emerges as the most prevalent mental health issue among elderly patients with somatic depressive symptoms, such as loss of energy, sleep disturbances and fatigue, moving into the foreground (Alexopoulos et al., 2002). Notably, the prevalence of manifest depressive symptoms seems to decline across age groups whereas the occurrence of minimal depressive symptoms appears to become more frequent in older individuals (Christl et al., 2025; Kessler et al., 2012). Therefore, minimal to mild or subthreshold depressive symptoms (SDS), that do not fulfil the criteria of a higher-order depressive disorder, may represent a prodrome of LLD and indicate a clinical high-risk state of depression (CHR-D) (Biella et al., 2019; Meisenzahl et al., 2024). These prevalences underscore the critical need for further research into depressive symptoms below the diagnostic threshold in elderly populations.

Subthreshold depressive symptoms exhibit a negative bidirectional relationship with psychosocial and cognitive functioning, physical well-being, and overall quality of life, thus constituting a significant burden of disability in older adults (Christl et al., 2025, 2026; Barry et al., 2009; Jeuring et al., 2016). For instance, SDS is associated with an increased risk for chronic medical conditions and mortality, including heightened suicide risk and a greater incidence of cardiovascular events (Alexopoulos, 2019; Fergusson et al., 2005; Sible et al., 2022). Conversely, the comorbidity of chronic somatic disorders and reduced physical functioning are significant risk factors for the progression into clinically manifest depressive disorders (Jeuring et al., 2016; Tuithof et al., 2018). Given the substantial burden of disability associated with SDS and the challenges related to its diagnosis and treatment, the identification of underlying biomarkers is critical for early detection of LLD and the development of targeted therapeutic strategies.

Resting-state functional magnetic resonance imaging (rs-fMRI) has the potential to offer objective insights into the neurophysiological underpinnings of psychiatric conditions. Both LLD and SDS have been linked to alterations in rs-fMRI derived functional connectivity (FC) within and between key brain networks (Gradone et al., 2023; Gandelman et al., 2019; Ely et al., 2016; Manning et al., 2019; Tan et al., 2023). These studies most frequently reported altered FC within the intrinsic default mode network (DMN) and between the DMN and other large-scale brain networks. The DMN is implicated in internally focused self-referential processes, enabling reflection on past experiences, emotions, and social interactions (Menon, 2023). Disruption of the DMN in depression has been linked to impaired emotion regulation and pathological rumination (Sheline et al., 2009; Hamilton et al., 2015). Other studies in elderly depressed patients with apathy, for instance, have reported reduced FC within the ventral attention network (VAN) (Yuen et al., 2014), a network whose functional expansion has recently been implicated as a potential risk factor and biomarker for depression (Lynch et al., 2024).

However, findings on functional network alterations in LLD and SDS remain heterogeneous, with studies reporting increased, decreased, or no detectable changes in FC (Saberi et al., 2022). These discrepancies may be attributed to small sample sizes (mostly *N* < 50) and methodological variability across study sites. Additionally, existing evidence is limited to cross-sectional analyses, leaving it unclear whether intra- and inter-network FC changes correspond with temporal variations in depressive symptom severity in older adults. In the context of psychosis, previous neuroimaging studies have demonstrated that FC abnormalities were already detectable during the CHR state, serving as biomarkers with a balanced classification accuracy of 72% (Hoheisel et al., 2024; Korda et al., 2022). Analogously, dimensional SDS may represent a CHR-state for LLD, yet its neurobiological underpinnings remain insufficiently characterized (Judd, 2012).

To address this gap, we analyzed the cross-sectional and longitudinal relationship between rs-fMRI derived FC and SDS in a large population-based sample of 243 healthy older adults at two time points. Specifically, FC was assessed within (intra-) and between (inter-) seven large-scale brain networks, including the DMN and VAN (Yeo et al., 2011). Given that aging is associated with alterations in functional network architecture, including a loss of network specialization, we also computed a network segregation score and examined its association with SDS (Chan et al., 2014; Stumme et al., 2020).

Although our analyses are exploratory in nature, based on well-powered studies in patients with LLD (Tan et al., 2023; Tozzi et al., 2021), as well as those in remitted LLD (Guan et al., 2022) we hypothesize a decrease in DMN FC associated with SDS. Additionally, given the prominence of cognitive and somatic symptoms in LLD, we expect that disconnection within the VAN will be associated with SDS in older adults.

## Materials and methods

### Participants

The present study was based on the 1000BRAINS project, a population-based study initially designed to investigate age-related functional and structural brain alterations and its correlation with behavioral, environmental and genetic factors (Caspers et al., 2014). Subjects were enrolled from the 10-year follow-up cohort of the epidemiological population-based Heinz Nixdorf Recall Study (HNR). Originally, no exclusion criteria except for MRI suitability were applied, which resulted in a sample of 969 older adults over the age of 55 years, of whom 369 underwent assessments at two time points (t0, t1). To exclude potential confounding variables, another 109 subjects were excluded at t0 due to the following reasons (see Supplementary Methods S1 for an overview of the exclusion process): Subjects with current or past psychiatric treatment, intake of antidepressants, moderate or severe depressive symptoms (BDI II > 19), subjects with neurological diseases that affect the central nervous system (e.g., stroke, multiple sclerosis, Parkinson’s disease, migraine, epilepsy, traumatic brain injury, brain tumor), and indication for severe cognitive impairment according to the dementia screening test DemTect (DemTect < 9) (Kalbe et al., 2004). To ensure optimal homogeneity of MRI analyses, another 17 subjects were excluded after visual image quality control.

All subjects gave written informed consent prior to inclusion in 1000BRAINS. The study protocol was in accordance with the Declaration of Helsinki and the study protocol was approved by the Ethics Committee of the University of Essen, Germany.

### MRI data acquisition

MRI acquisition was thoroughly described in a previous paper by Caspers et al. (2014) with a detailed description of the 1000BRAINS study protocol. Briefly, MRI was performed on a 3 T Siemens Tim-TRIO scanner with a 32-channel head coil (Erlangen, Germany). Rs-fMRI data was acquired as a blood-oxygen level-dependent (BOLD) gradient-echo planar imaging (EPI) sequence with 36 transversely oriented slices with the following parameters: slice thickness 3.1 mm, TR = 2,200 ms, TE = 30 ms, FoV = 200 × 200 mm^2^, voxel resolution 3.1 mm× 3.1 mm× 3.1 mm. Subjects were instructed to keep their eyes closed, let their mind wander, and do not fall asleep. The acquisition lasted approximately 11 min producing 300 volumes.

### Functional image preprocessing

Image preprocessing was conducted using the FSL toolbox (FMRIB Software Library^1^) (Jenkinson et al., 2012). Initially, the first four EPI volumes of each subject were discarded. Functional images were then corrected for head motion using rigid-body registration. Subsequently, all volumes were aligned to the first image on which a mean image was created, providing the basis to which all volumes were aligned. ICA-based Automatic Removal Of Motion Artifacts (ICA-AROMA) (Pruim et al., 2015), combined with global signal regression (Burgess et al., 2016; Ciric et al., 2017; Parkes et al., 2018), was employed to eliminate motion-related independent components from the fMRI data. Next, images were bandpass filtered (0.001–0.1 Hz) and normalized to the MNI152 standard space template using the unified segmentation approach (Ashburner and Friston, 2005). We further applied the algorithm by Afyouni and Nichols (2018) to identify severe intensity dropouts by generating *p*-values for spikes (DVARS) on the preprocessed data, excluding subjects with more than 10% corrupted spikes across the 300 volumes. Finally, we checked for potential misalignments of the preprocessed mean AROMA data to the MNI152 template (deviation > 2 SD) using the “check sample homogeneity using standard deviation across sample” function provided by the CAT12 toolbox (Gaser et al., 2024).

### Functional connectivity parameters

The cortical parcellation of Schaefer et al. (2018) was used to analyze FC within established cortical networks. Cortical network parcellation was performed by clustering the whole-brain FC regarding their similarity of functional activation profiles which leads to a parcellation of 400 different regions allocated to seven functional networks, namely visual- (VN), sensorimotor- (SMN), limbic- (LN), control- (CN), default mode- (DMN), dorsal- (DAN) and ventral attention networks (VAN), as defined by Yeo et al. (2011).

For FC network analyses, graph-theoretical parameters were computed by converting individual functional data into whole-brain graphs (i.e., connectomes; Rubinov and Sporns, 2010), with each region of interest (ROI) represented by a mean BOLD time series comprising 296 time points (excluding the first four volumes). Node-wise mean time series were extracted from the preprocessed rs-fMRI data using fslmeants (Smith et al., 2004), averaging the time series of all voxels corresponding to each node. Functional connections between nodes (i.e., edges) were quantified using Pearson’s product–moment correlation of the respective average BOLD time series, yielding a symmetric 400 × 400 matrix (according to the Schaefer parcellation), where each entry represents a Pearson correlation coefficient between two nodes.

To reduce noise-related edges, we incorporated the statistical significance of each Pearson correlation coefficient as an additional preprocessing step. The observed time series were randomized by performing a Fourier transform, scrambling the phase, and then inverting the transform (Stumme et al., 2020; Zalesky et al., 2012). This process was repeated 1,000 times, followed by a permutation test, setting non-significant edges at *p* ≥ 0.05 to zero. The resulting adjacency matrix was transformed into z-scores using Fisher’s *r*-to-*z* transformation, including both positive and negative correlations. Given that including both positive and negative weights in the estimation of strength values might cause mutual suppression, we performed separate estimations: one for positive correlations (FC_pos_) and another for negative correlations (FC_neg_), setting the opposite correlations to zero in each case.

Each of the FC_pos_ and FC_neg_ whole brain connectomes (400 × 400 connectivity matrices) were then transformed into a triangular matrix (diagonal set to NaN) as only unidirectional information of edges was used. Based on the two different matrices, we calculated three different parameters for each node:

- Intra-network connectivity as the sum of weights (i.e., connectivity values) of edges from one node to all nodes within its corresponding network divided by the number of all edges comprising the network (for n nodes, there are n*[n-1]/2 possible edges in a fully connected network)
- Inter-network connectivity as the sum of weights of edges from one node to all nodes outside its corresponding network divided by the number of the respective edges of the network
- Between-network connectivity as the sum of weights of edges between all respective nodes in two different networks divided by the number of the respective edges.

Additionally, as a measure of altered network architecture we computed the network segregation score (*Q*) as a combined quantitative ratio integrating both, the intra- as well as the inter-network connectivity as follows:

$$Q=\frac{\text{intra}-\text{network connectivity}-\text{inter}-\text{network connectivity}\;}{\text{intra}-\text{network connectivity}+\text{inter}-\text{network connectivity}}$$

The segregation score was initially employed by Chan et al. (2014) and refined by Stumme et al. (2020). Specifically, a score of 1 represents maximal segregation (high intra- and low inter-network connectivity), a score of −1 implies maximal network integration (low intra- and high inter-network connectivity). A score of 0 indicates a balanced system. Loss of network segregation, for instance, has been previously observed in aging populations (Stumme et al., 2020; Jockwitz and Caspers, 2021).

Thus, for each node, six different strength values were calculated: Two intra-network, two inter-network and two between-network estimates (for FC_pos_ and FC_neg_ respectively) resulting in 2400 connectivity values (400 nodes x 6 strength values) for each subject plus the network segregation score (Q). Statistical analyses were initially performed on network level.

### Depression measures

Depressive symptom severity was assessed with the Beck Depression Inventory II (BDI-II) (Beck et al., 1996; Hautzinger et al., 2006). Participants with BDI-II scores higher than 19 (which indicate moderate or severe depression) were excluded. Additionally, we subdivided the BDI-II score into three symptom domains: cognitive, affective, and somatic, to determine which domain primarily drives the overall association (Almeida et al., 2023). The average BDI-II score was 4.05 (±3.58) at t0 and 4.14 (±3.74) at t1 (see Table 1).

**Table 1:** Descriptive statistics of the included subjects at t0 and t1.

Variables	t0	t1
*N* =	243	243
Age, years	67.05 (±6.55)	70.79 (±6.53)
ISCED 97	6.76 (±1.93)	6.77 (±1.93)
Psychotropic drug intake	0 (0.0)	2 (0.8)
BDI-II	4.05 (±3.58)	4.14 (±3.74)
DemTect	14.79 (±2.35)	14.87 (±2.54)

^1^Numbers present either absolute numbers plus percent or mean plus standard deviation, ISCED 97 = International Standard Classification of Education 97, BDI-II = Beck Depression Inventory II, DemTect = screening test for dementia, t0 = baseline assessment, t1 = follow-up assessment.

### Statistics

To unveil FC network markers of subthreshold depressive symptom severity in older adults, we performed cross-sectional as well as longitudinal analyses. All analyses were performed with IBM SPSS Statistics 27 (IBM, C, 2020). Results for FC_neg_ will be presented in the Supplementary Results S1.

First, we computed cross-sectional linear regression models to examine the relationship between depressive symptoms and all FC measures at the first time point (t0). Second, we investigated how longitudinal changes in depressive symptom severity were associated with alterations in FC measures. We calculated *Δ*BDI-II scores (ΔBDI-II = BDI-II_t1_ – BDI-II_t0_), with positive Δ-values indicating an increase in depressive symptoms over time. Repeated-measures analyses of covariance (ANCOVA) were then performed, using the FC measures at t0 and t1 as within-subject variables (time), with sex as a between-subject factor and age, educational level, time between scans, and ΔBDI-II as covariates.

Third, we analyzed whether baseline network FC (at t0) was associated with longitudinal changes in depressive symptom severity. Specifically, we hypothesized that decreased network connectivity at t0 would predict a deterioration in depressive symptoms. To test this, we computed repeated-measures ANCOVA with BDI-II scores at t0 and t1 as within-subject variables (time), sex as a between-subject factor and age, educational level, time between scans, and the respective FC measure at t0.

The second and third approach were chosen to address complementary longitudinal questions: first, whether changes in depressive symptoms are associated with concurrent changes in functional connectivity over time (time × ΔBDI-II interaction), and second, whether disruptions in functional network architecture at baseline predict subsequent worsening of depressive symptoms (time × FC(t0) interaction).

For all analyses, we included age, sex, and education, as classified by the International Standard Classification of Education 97 (ISCED 97; UNESCO, 2006), as covariates. Additionally, time between scans was included as a covariate in all longitudinal analyses.

To account for multiple comparisons, we applied the Bonferroni correction by dividing the significance threshold by the number of functional networks analyzed (*p*-corrected = 0.05/7 = 0.007). Consequently, results were considered statistically significant if the *p*-value of an analysis was less than 0.007.

Finally, for all significant associations between FC and SDS, we calculated FC within each ROI in both the left and right hemispheres comprising the network (for information about the respective ROIs that constitute a network^2^). We then identified the specific ROIs driving the observed significant global association. We applied the Benjamini-Hochberg procedure to correct for multiple comparisons (Benjamini and Hochberg, 1995).

## Results

### Sociodemographic and clinical statistics

In total, the present study consists of *N* = 243 subjects (aged 55–84 years) at t0 and t1. The mean time between visits was 3.7 years (SD = 0.7). See Table 1 for sociodemographic details. Participants did not differ regarding ISCED97 (*T*(484) = −0.047, *p* (two-sided) = 0.963, 95% CI [−0.353, 0.336]), cognitive functioning (*T*(484) = −0.310, *p* (two-sided) = 0.757, 95% CI [−0.574, 0.418]) and psychotropic drug intake (*T*(242) = −1.417, *p* (two-sided) = 0.158, 95% CI [−0.020, 0.003]) between the two assessments. BDI-II values between assessments also did not differ significantly (*T*(484) = −0.298, *p* (two-sided) = 0.766, 95% CI [−0.751, 0.554]), instead they were highly correlated controlling for age, sex, education and time between assessments (*r* = 0.568, *p* < 0.001).

### Results from cross-sectional analyses

**Figure 1:**
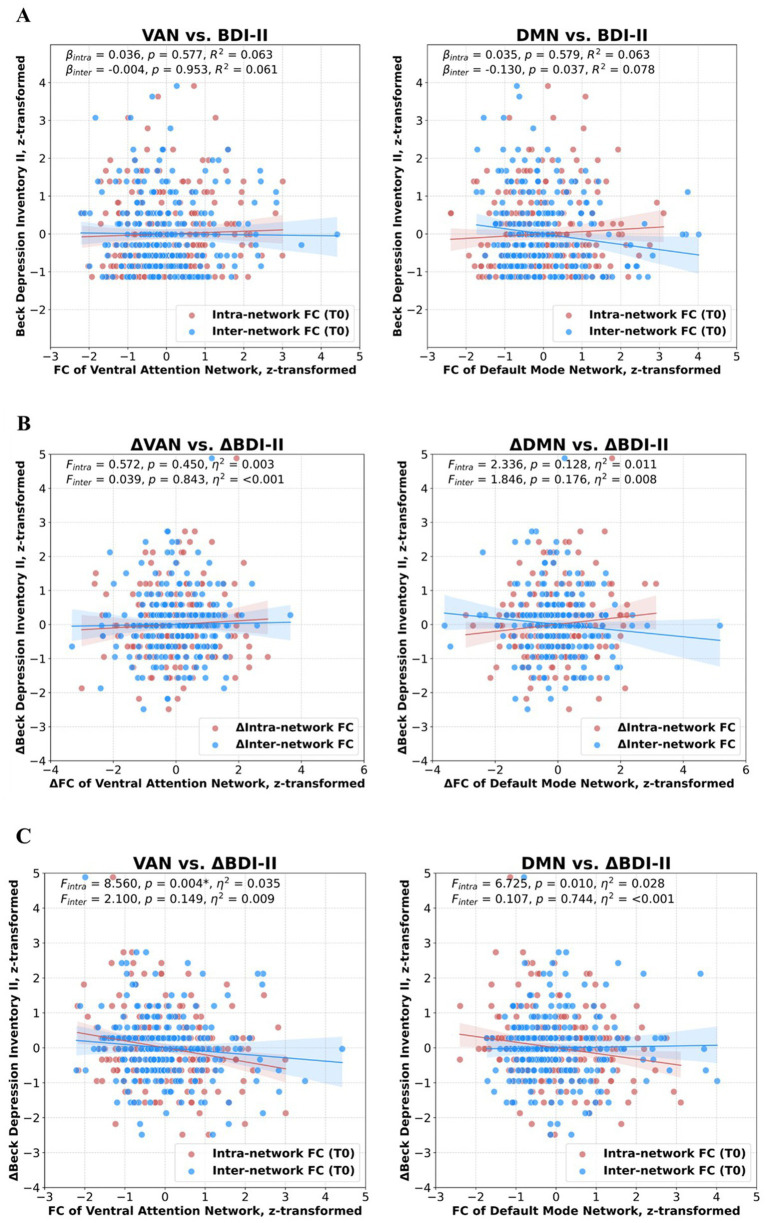
Scatterplots illustrating the relationship between positive intra-network as well as inter-network functional connectivity (FC) of the ventral attention network (VAN) and default mode network (DMN) with Beck Depression Inventory II (BDI-II) scores. All values were *z*-transformed. **(A)** Cross-sectional relationship between FC of the three networks and BDI-II scores at the first visit (t0). **(B)** Longitudinal relationship between changes in FC (ΔFC = FC_t1_ – FC_t0_) and changes in BDI-II (ΔBDI-II = BDI-II _t1_ – BDI-II _t0_). **(C)** Prediction of ΔBDI-II using FC of the three networks at t0. The relationship between FC within the VAN at t0 and ΔBDI-II remained significant after Bonferroni correction (*p* 0 < 0.007).

**Figure 2:**
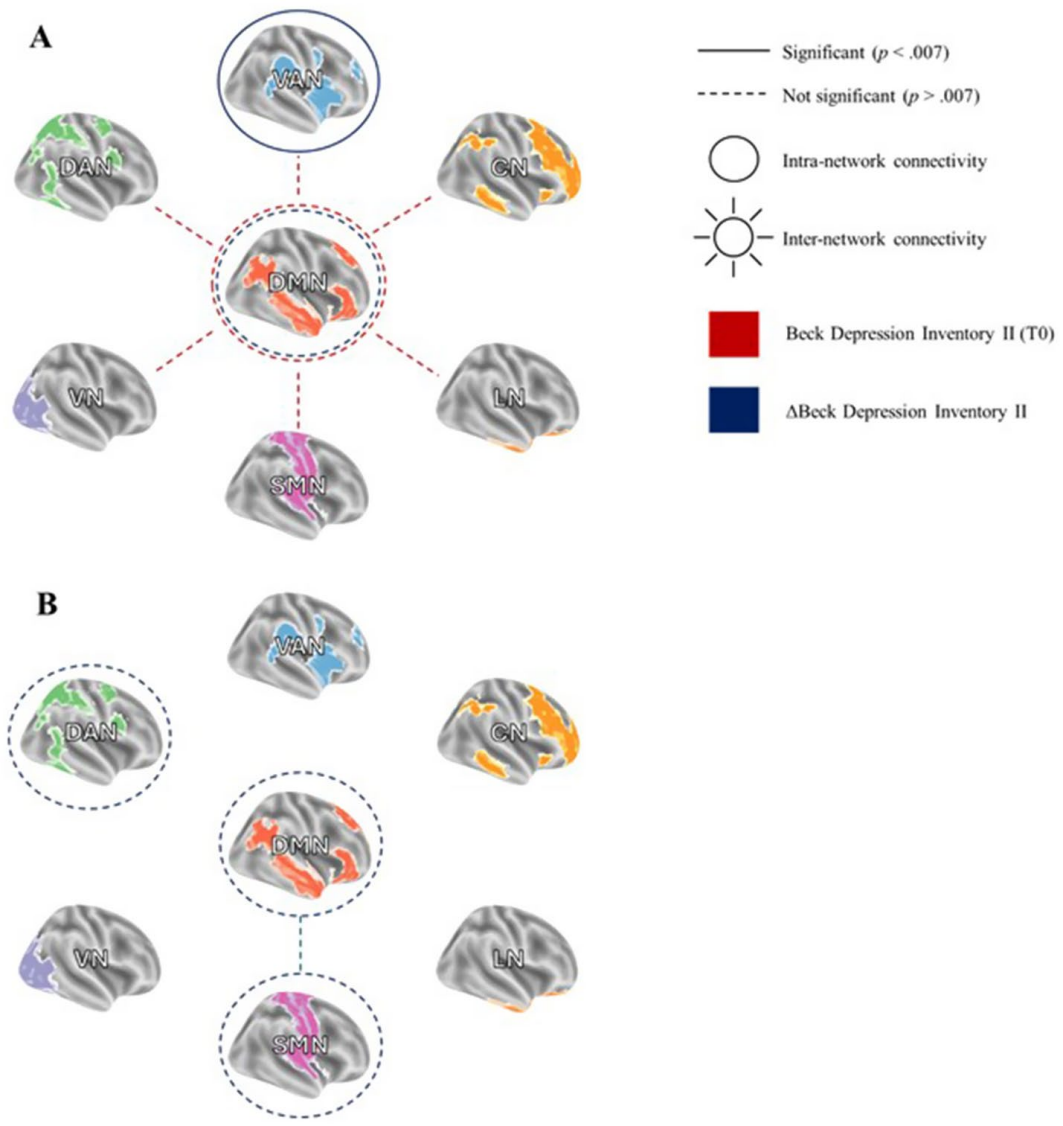
Reduced positive intra- and inter-network connectivity related to subthreshold depressive symptom severity (Beck Depression Inventory II, BDI-II). **(A)** Lower inter-network functional connectivity (FC) of the default mode network (DMN) is associated with higher BDI-II scores at the first visit (t0). Additionally, reduced intra-network FC within the DMN and ventral attention network (VAN) at t0 is linked to changes in depressive symptoms (ΔBDI-II = BDI-II _t1_ – BDI-II _t0_). **(B)** Longitudinal changes in intra-network FC within the dorsal attention network (DAN) and FC between the DMN and somatomotor network (SMN) are associated with ΔBDI-II. VAN = ventral attention network, CN = control network, LN = limbic network, SMN = somatomotor network, VN = visual network, DAN = dorsal attention network, DMN = default mode network.

At t0, the inter-network connectivity of the DMN decreased with increasing SDS (*R*^2^ = 0.078, *β* = −0.130, *p* = 0.037; see Figures 1, 2A), however, it did not remain significant after applying the Bonferroni correction for multiple testing. Moreover, we observed a positive trend-level association of SDS with DMN segregation (*p* = 0.067). For an overview of all regression analyses, please see Supplementary Results S2.

### Results from longitudinal analyses

Conducting repeated-measures ANCOVA, we observed a significant negative time x ΔBDI-II interaction effect for longitudinal alterations of intra-network FC_pos_ of the DAN [*F*(1, 222) = 4.248, *p* = 0.040, partial η^2^ = 0.019], and FC_pos_ between the SMN-DMN [*F*(1, 222) = 4.386, *p* = 0.037, partial η^2^ = 0.020; see Figure 2B]. Moreover, analyses revealed a positive interaction effect for DMN segregation [*F*(1, 222) = 3.928, *p* = 0.049, partial η^2^ = 0.018]. However, these associations did not withstand the Bonferroni correction. There was no significant interaction effect for any inter-network FC_pos_ measure (please see Supplementary Results S3 for all analyses).

### Associations of baseline functional connectivity with longitudinal BDI-II alterations

There were significant time × FC_pos_ (t0) interaction effects for the VAN and the DMN: Longitudinal increasing depressive symptoms were associated with reduced baseline intra-network FC_pos_ [*F*(1, 241) = 8.560, *p* = 0.004, partial η^2^ = 0.035] and segregation of the VAN [*F*(1, 241) = 4.141, *p* = 0.043, partial η^2^ = 0.017], as well as baseline intra-network FC_pos_ [*F*(1, 241) = 6.725, *p* = 0.010, partial η^2^ = 0.028] and segregation of the DMN [*F*(1, 241) = 4.255, *p* = 0.040, partial η^2^ = 0.018]. The association with baseline intra-network FC_pos_ of the VAN remained significant after applying the Bonferroni correction (see Figures 1, 2A and Supplementary Results S4).

To prove whether baseline FC_pos_ within the VAN provides predictive information beyond baseline symptom severity, we performed a multiple regression analysis with VAN- FC_pos_ as the independent variable and ΔBDI-II as the dependent variable controlling for age, sex, ISCED97, time between scans and baseline BDI-II. This analysis showed that after adding baseline BDI-II as a covariate, the association between VAN- FC_pos_ and ΔBDI-II remains significant (*T* = −2.98, *p* = 0.003, *β* = −0.173, adjusted *R*^2^ = 0.217).

Additional robustness analyses revealed, that the observed significant association between baseline VAN-FC and ΔBDI-II remained significant after excluding participants with mild depressive symptoms [*n* = 5; *F*(1, 232) = 7.643, *p* = 0.006, partial η^2^ = 0.032] and participants without any depressive symptoms at baseline [*n* = 36; *F*(1, 201) = 6.214, *p* = 0.013, partial η^2^ = 0.030].

The significant association of baseline intra-network FC of the VAN and longitudinal BDI-II changes was mainly driven by the somatic symptom domain [*F*(1, 237) = 9.667, *p* = 0.002, partial η^2^ = 0.039], in contrast to the cognitive [*F*(1, 237) = 2.258, *p* = 0.134, partial η^2^ = 0.009] and affective domains [*F*(1, 237) = 0.276, *p* = 0.600, partial η^2^ = 0.001].

### *Post-hoc* analyses within ventral attention network regions

**Figure 3:**
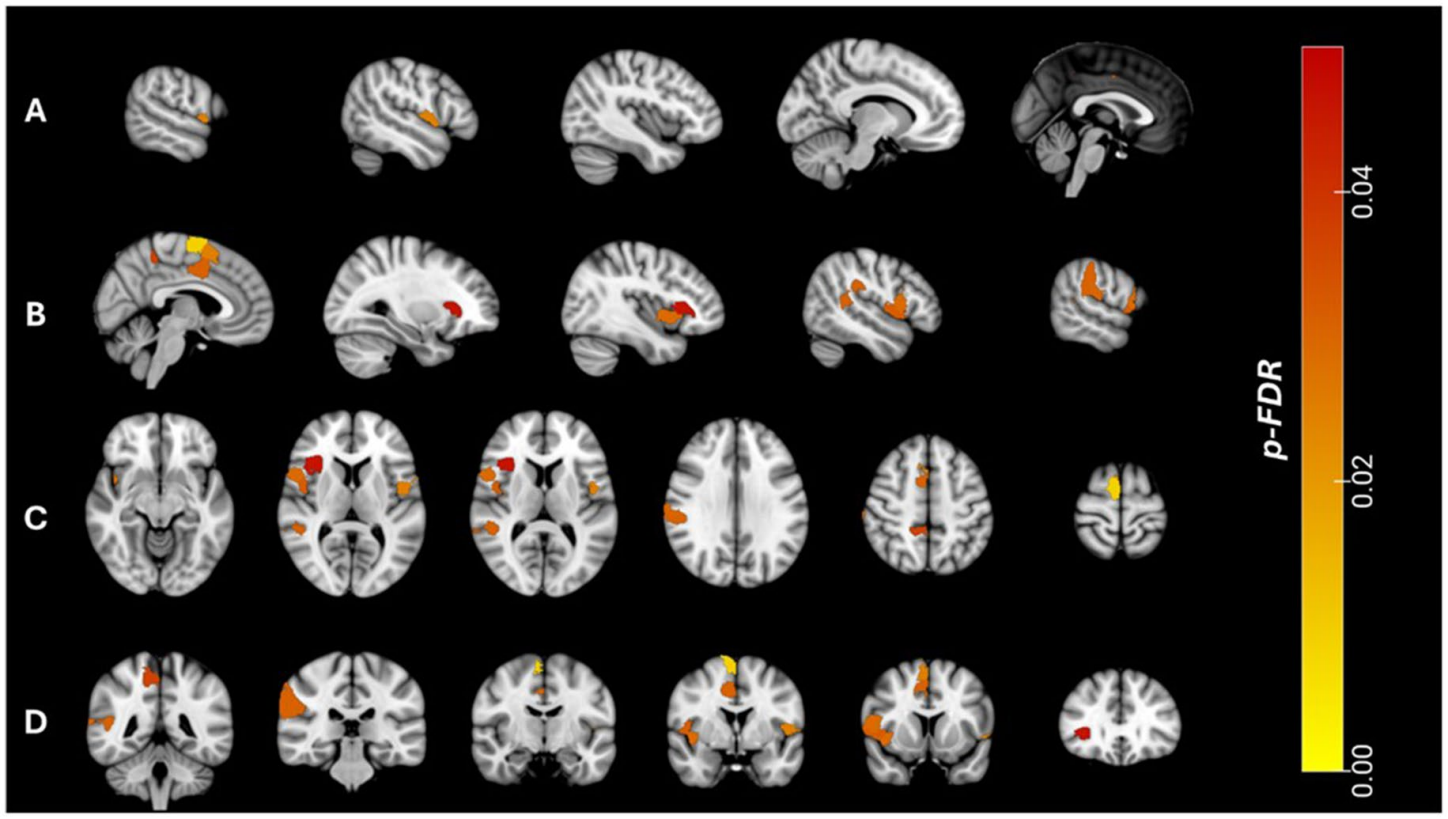
Associations between reduced positive intra-network functional connectivity (FC_pos_) within regions of interest (ROIs) of the ventral attention network (VAN) and longitudinal changes in subthreshold depressive symptoms (ΔBeck Depression Inventory II, ΔBDI-II = BDI-II_t1_ – BDI-II_t0_) using the 7-network Schaefer parcellation (400 nodes). The heatmap represents the FDR-corrected *p*-values for the association between each ROI and ΔBDI-II scores. **(A)** Sagittal view, left hemisphere. **(B)** Sagittal view, right hemisphere. **(C)** Axial view. **(D)** Coronal view.

*Post-hoc* analyses revealed a significant interaction effect between BDI-II changes over time and FC_pos_ (t0) for left hemispheric VAN connectivity [*F*(1, 237) = 6.567, *p* = 0.011, partial η^2^ = 0.027] as well as right hemispheric VAN connectivity [*F*(1, 237) = 9.338, *p* = 0.003, partial η^2^ = 0.038]. The significant global association with VAN connectivity was mainly driven by right hemispheric FC_pos_ in temporo-occipital-parietal regions, the frontal operculum and insula as well as medial parietal regions (see Figure 3 and Supplementary Results S5).

## Discussion

To our knowledge, this study is the first to examine both cross-sectional and longitudinal relationships between SDS and rs-fMRI derived FC alterations within and between seven large-scale functional brain networks defined by Yeo et al. (2011). We analyzed a well-characterized cohort of 243 healthy older adults without a history of psychiatric treatment or current antidepressant use. Our findings demonstrate that even depressive symptoms (BDI-II < 20) that do not meet the criteria for higher-order depression, are associated with reduced FC, particularly within and between the intrinsic DMN as well as the extrinsic VAN – although most associations did not stay significant after applying the conservative Bonferroni correction. However, a link between decreased intra-network FC within the VAN at baseline and longitudinal increasing depressive symptoms remained significant. Moreover, this association was mainly driven by right hemispheric parcels within the VAN as well as the somatic symptom domain of the BDI-II. *Post-hoc* regression analyses revealed that VAN-FC provides predictive information beyond baseline symptom severity proving the added utility of FC as a biomarker.

Taken together, these findings support the hypothesis that disruptions in functional network organization precede the emergence of depressive symptoms in later life and may thus mark the transition into clinically manifest LLD. This underscores the relevance of a dimensional framework for understanding depressive states in aging populations (Biella et al., 2019; Meisenzahl et al., 2024; Judd, 2012; Benasi et al., 2021). The observed FC changes may serve as early neural markers and could help in the early prediction and intervention of individuals at risk, especially in the context of aging populations with rising SDS prevalence (Christl et al., 2025; Pietrzak et al., 2013).

### The ventral attention network in the emergence of depressive symptoms

We observed longitudinal changes in depressive symptoms to be associated with altered FC of the VAN at baseline, in particular within temporo-occipital-parietal parcels, the frontal operculum and insula as well as medial parietal regions of the right hemisphere. This suggests that disconnection of these parcels within the VAN could predict a deterioration of depressive symptoms. The VAN, also known as the salience network, plays a crucial role in directing attention based on the novelty or unexpectedness of stimuli. It functions in a bottom-up manner to prioritize salient stimuli, thereby effectively interrupting the DMN (Vossel et al., 2014). In other words, the salience network combines conscious integration of automatic feedback and responses to environmental demands and personal goals (Seeley, 2019). Given that emotional stimuli possess high salience, they are more likely to capture attention. Individuals with LLD or SDS often exhibit a bias towards negative emotional stimuli, such as during facial recognition tasks (Baruch et al., 2021), which also seems to be a possible behavioral predictor for symptom remission (Mei et al., 2020).

In line with these behavioral observations, a task-based fMRI study in subjects with LLD demonstrated lower FC between the salience network and other networks during a cognitive control task with emotional stimuli (Almdahl et al., 2023). Consistent with our finding’s, reduced FC has also been reported for the right hemispheric salience network, suggesting that alterations within the VAN may already be detectable in the prodromal state of LLD and may progress with increasing symptom severity. Additionally, these findings extend prior cross-sectional findings (Gradone et al., 2023; Tan et al., 2023) by providing longitudinal evidence that VAN dysconnectivity is linked to the emergence of depressive symptoms and may precede the clinical transition into LLD. The VAN plays a homeostatic role by modulating the dynamic balance between the intrinsic DMN and the executive CN (also referred to as the frontoparietal network) (Goulden et al., 2014). Dysfunction of these large-scale functional networks has led to the declaration of a network-based model of LLD (Gunning et al., 2021; Menon and Uddin, 2010). Thus, dysfunction of the VAN is characterized by increased salience attribution to negative stimuli and decreased salience to positive stimuli. In conjunction with disrupted DMN connectivity, this imbalance may reflect a maladaptive shift between internal and external emotion regulation strategies. As a result, reduced intra-network FC of the VAN may contribute to diminished motivation and loss of interest in external stimuli. Supporting this, Yuen et al. (2014) observed decreased FC within the salience network as well as between the salience network and the executive CN in elderly depressed patients with high comorbid apathy – a condition of insensitivity to external stimuli – compared to those with lower apathy. Our own *post-hoc* analyses revealed that the overall significant association was mainly driven by changes in the somatic symptom domain of the BDI-II. This aligns with clinical observations of LLD, with somatic symptoms such as concentration difficulties, loss of energy, fatigue, etc., moving into the foreground (Alexopoulos et al., 2002). In retrospection, the majority of patients reported about somatic complaints in the prodromal phase of depression (Meisenzahl et al., 2024). In addition, retrospective analysis of medical records supports a somatic presentation before onset of depression (Cadoret et al., 1980; Wilson et al., 1983). Low rates of recognition in primary care could be explained by this somatic form of presentation (Kapfhammer, 2006). In conclusion, our results support the importance of somatic symptoms in SDS and its potential association with other biomarkers such as FC of the VAN.

Building on recent discussions, the salience network has recently garnered increased attention, as Lynch et al. (2024) reported robust functional cortical expansion of this network in individuals with depression. In a subset of five older participants who underwent repeated clinical assessments and fMRI scans, this pattern was replicated, suggesting an age-independent effect. Notably, the same expansion was observed in children prior to the onset of depression in later adulthood, indicating its potential as the first early predictive marker for depression. In line with this, greater cortical thickness of the right insula — a key region within the VAN — has been associated with reductions in apathy following antidepressant pharmacotherapy in LLD (Pimontel et al., 2021). Additionally, subjects with LLD and reduced salience network activation have shown the greatest therapeutic benefit from psychotherapy (Solomonov et al., 2024). Functional alterations within this network also appear to have transdiagnostic relevance, distinguishing depression from schizophrenia (Huang et al., 2022). Collectively, these findings suggest that VAN dysfunction may not only mark increasing depressive symptoms and facilitate early detection of subjects at high risk, but also inform targeted early intervention strategies (Alexopoulos, 2019; Karim et al., 2017; Zheng et al., 2020).

### Disruption of the default mode network and the link to subthreshold depressive symptoms

Our findings revealed that reduced inter-network FC of the DMN at baseline (t0) was associated with higher depressive symptoms. Furthermore, participants exhibiting decreased intra-network FC at t0 were more susceptible to increasing depressive symptoms over time, though these results did not withstand the Bonferroni correction. The DMN, which is active during self-referential cognition and episodic memory retrieval, is implicated in self-perception and social interactions (Menon, 2023). It has been extensively studied in the context of depression pathophysiology (Kaiser et al., 2015), particularly in relation to rumination (Hamilton et al., 2015) and the regulation of negative emotions (Martins and Mather, 2016).

Previous rs-fMRI studies have identified heterogeneous patterns of abnormal FC in the DMN associated with SDS in older adults. Some studies report hyperconnectivity (Ely et al., 2016), others report hypoconnectivity (Gradone et al., 2023; Zhu et al., 2014), and some report both (Li et al., 2014; Philippi et al., 2015). Additionally, Touron et al. (2022) found PET-derived glucose hypometabolism in DMN and fronto-limbic regions among individuals with SDS. However, more extensive research has examined FC alterations in clinically manifest LLD. These studies also report inconsistent findings, with some indicating hyperconnectivity (Alexopoulos et al., 2012; Eyre et al., 2016), hypoconnectivity (Gandelman et al., 2019; Tan et al., 2023), or both (Li et al., 2017) within the DMN. In a well-powered sample of 416 subjects, Tan et al. (2023) showed decreased intra-network FC most prominent within the DMN and the SMN in subjects with LLD compared to healthy controls. Furthermore, Guan et al. (2022) observed decreased FC of the DMN in remitted LLD subjects compared to controls. A meta-analysis by Saberi et al. (2022) reported no significant FC alterations between LLD and healthy controls. The authors attributed the heterogeneous results across studies to factors such as small sample sizes and methodological variability. Our cross-sectional and longitudinal findings point in the direction that DMN disconnection from other networks and hypoconnectivity within the DMN may indeed be potential neural markers of increasing depressive symptoms in older adults. For example, Gerlach et al. (2025) demonstrated that lower connectivity of the DMN to the salience network was associated with relapse in recently remitted subjects with LLD. While our associations did not remain significant after correction for multiple comparisons, the observed patterns are consistent with prior reports and underscore the potential relevance of DMN dysconnectivity as a target for further investigation.

### Limitations

In addition to various methodological strengths, such as the relatively large sample size and the combination of cross-sectional and longitudinal analyses, this study has also some limitations. First, the use of the BDI-II, a self-report instrument, restricts our analysis to subjectively reported depressive symptoms, although BDI-II demonstrates high internal consistency in older adults (Balsamo et al., 2018). Second, our representative, population-based sample, encompassing a broad age range, includes individuals with diverse biographical, psychological, and medical characteristics. Future subtyping studies will be essential to delineate distinct neuro-phenotypes within this heterogeneity and to advance personalized approaches to risk prediction and intervention (Tozzi et al., 2024). More fundamentally, LLD represents a clinically and biologically heterogenous condition, suggesting that distinct neurobiological pathways may ultimately converge to produce similar depressive phenotypes (Dukart et al., 2024), thereby complicating the interpretation of underlying neurophysiological mechanisms. Integrating data across neuroimaging modalities (including neurotransmitter mapping) may offer a promising approach for disentangling this complexity in future research (Lotter and Dukart, 2025). Taken together, future longitudinal studies with large, heterogeneous clinical populations are necessary to identify consistent neuroimaging biomarkers for biologically distinct LLD phenotypes (Saberi et al., 2022).

## Conclusion

In conclusion, our results highlight the critical association between reduced FC within the right-hemispheric VAN and longitudinally increased depressive symptoms, suggesting its potential as an early neural marker of transition into LLD. This association was predominantly driven by the somatic symptom domain of the BDI-II, consistent with clinical features commonly observed in LLD. Given the elevated risk of dementia and cardiovascular events associated with LLD, our findings underscore the clinical relevance of early detection and intervention strategies in individuals at clinically high risk.

## Data Availability

The datasets presented in this article are not readily available because restrictions apply. Data are, however, available from the authors upon reasonable request and with permission from Svenja Caspers. Requests to access the datasets should be directed to julia.christl@lvr.de.

## Footnotes

1
http://www.fmrib.ox.ac.uk/fsl

2
https://github.com/ThomasYeoLab/CBIG/tree/master/stable_projects/brain_parcellation/Schaefer2018_LocalGlobal/Parcellations/MNI
