# Supplementary material for: Cross-sectional and longitudinal functional network alterations associated with subthreshold depressive symptoms in healthy older adults
Source: Front Aging Neurosci. 2026 Mar 11;18:1742371. doi: 10.3389/fnagi.2026.1742371 (PMC13023060; doi:10.3389/fnagi.2026.1742371)
Supplement: Supplementary file 1 [file Data_Sheet_1.docx]

**Supplementary Material**

**Supplementary Methods S1: Exclusion process**

In total, N = 969 individuals aged 55 years and older were enrolled from the 1000BRAINS cohort; 369 of these individuals participated in two assessments. In 1000BRAINS, the only exclusion criterion was suitability for magnetic resonance imaging. However, to obtain a sample comprising individuals with only subclinical depressive symptoms and no manifest psychiatric conditions, in the present study we excluded individuals with current or past psychiatric treatment, antidepressant intake, moderate or severe depressive symptoms (Beck Depression Inventory II [BDI-II] score > 19), neurological diseases that affect the central nervous system, or an indication of severe cognitive impairment according to the dementia screening test DemTect (DemTect score < 9) (see Supplementary Figure S1). Moreover, we excluded individuals with a preprocessing failure in the functional imaging data. Our dataset therefore comprised N = 243 older adults with no moderate or severe depressive symptoms.


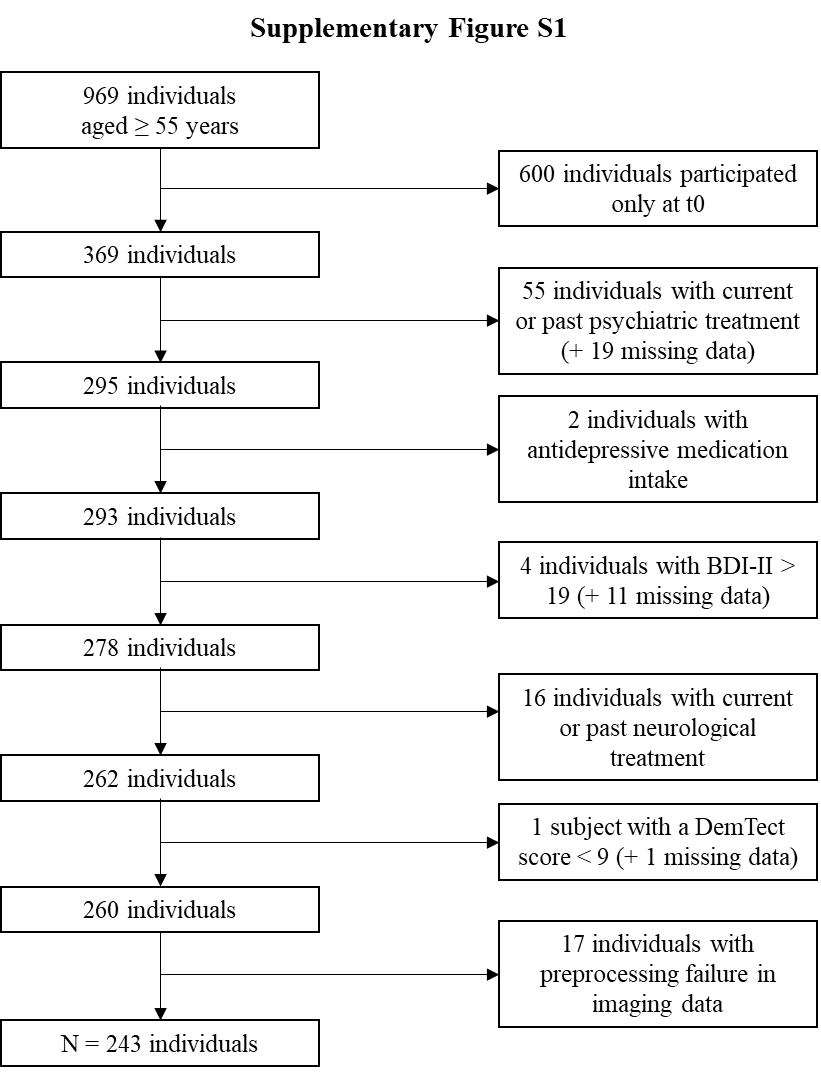


**Supplementary Results S1: Detailed results from cross-sectional analyses**

Results are from cross-sectional linear regression models with subthreshold depressive symptoms (SDS; measured with the BDI-II and defined as a BDI-II score < 20) as the dependent variable and intra- and inter-network FC_pos_ and network segregation as independent variables. Corrections were made for age, sex, and educational level (International Standard Classification of Education 1997).

We found a significant negative association between SDS and the inter-network FC_pos_ of the DMN (*p* = .037), but this association was no longer significant after Bonferroni correction. See Supplementary Tables S1 and S2 for detailed results.

| **Supplementary Table S1** Linear regression analyses for subthreshold depressive symptoms versus positive functional connectivity at t0* | | | | | |
| --- | --- | --- | --- | --- | --- |
| Independent variables | B | SE | *β* | *p* | R² |
| Visual network (intra) | -0.586 | 1.615 | -0.023 | 0.717 | 0.062 |
| Visual network (inter) | -0.350 | 5.451 | -0.004 | 0.949 | 0.061 |
| Visual network (segregation) | -0.692 | 1.636 | -0.027 | 0.673 | 0.062 |
| Somatomotor network (intra) | -0.674 | 1.654 | -0.026 | 0.684 | 0.062 |
| Somatomotor network (inter) | 0.317 | 6.260 | 0.003 | 0.960 | 0.061 |
| Somatomotor network (segregation) | -2.530 | 2.152 | -0.075 | 0.241 | 0.067 |
| Dorsal attention network (intra) | 2.403 | 3.196 | 0.047 | 0.453 | 0.064 |
| Dorsal attention network (inter) | -1.677 | 7.616 | -0.014 | 0.826 | 0.062 |
| Dorsal attention network (segregation) | 1.728 | 2.082 | 0.052 | 0.407 | 0.064 |
| Ventral attention network (intra) | 1.569 | 2.806 | 0.036 | 0.577 | 0.063 |
| Ventral attention network (inter) | -0.581 | 9.818 | -0.004 | 0.953 | 0.061 |
| Ventral attention network (segregation) | 1.354 | 2.206 | 0.039 | 0.540 | 0.063 |
| Limbic network (intra) | -3.488 | 3.040 | -0.073 | 0.252 | 0.067 |
| Limbic network (inter) | -20.972 | 10.956 | -0.119 | 0.057 | 0.076 |
| Limbic network (segregation) | 0.061 | 1.686 | 0.002 | 0.971 | 0.061 |
| Control network (intra) | -7.101 | 3.891 | -0.113 | 0.069 | 0.074 |
| Control network (inter) | -9.903 | 12.538 | -0.049 | 0.430 | 0.064 |
| Control network (segregation) | -2.114 | 2.012 | -0.066 | 0.295 | 0.066 |
| Default mode network (intra) | 2.769 | 4.990 | 0.035 | 0.579 | 0.063 |
| **Default mode network (inter)** | **-24.255** | **11.565** | **-0.130** | **0.037** | **0.078** |
| Default mode network (segregation) | 3.900 | 2.120 | 0.115 | 0.067 | 0.075 |
| *The dependent variable was the Beck Depression Inventory II score at the first visit (t0), and the covariates were age, sex, and International Standard Classification of Education 1997 (ISCED97) level.  B, regression coefficient; β, standardized regression coefficient; inter, inter-network positive functional connectivity; intra, intra-network positive functional connectivity; R^2^, adjusted coefficient of determination; SE, standard error | | | | | |

| **Supplementary Table S2** Linear regression analyses for subthreshold depressive symptoms versus positive functional connectivity between networks at first visit (t0) | | | | | |
| --- | --- | --- | --- | --- | --- |
| Independent variables | B | SE | *β* | *p* | R² |
| Visual↔somatomotor network | 1.047 | 1.782 | 0.037 | 0.558 | 0.063 |
| Visual↔dorsal attention network | -0.958 | 2.719 | -0.022 | 0.725 | 0.062 |
| Visual↔ventral attention network | 1.654 | 5.215 | 0.020 | 0.751 | 0.062 |
| Visual↔limbic network | -0.754 | 5.375 | -0.009 | 0.889 | 0.062 |
| Visual↔control network | -6.794 | 7.887 | -0.054 | 0.390 | 0.064 |
| Visual↔default mode network | -11.300 | 7.179 | -0.098 | 0.117 | 0.071 |
| Somatomotor↔dorsal attention network | 0.490 | 2.887 | 0.011 | 0.865 | 0.062 |
| Somatomotor↔ventral attention network | 0.586 | 3.499 | 0.010 | 0.867 | 0.062 |
| Somatomotor↔limbic network | -7.619 | 5.196 | -0.093 | 0.144 | 0.070 |
| Somatomotor↔control network | 5.089 | 7.198 | 0.044 | 0.480 | 0.063 |
| Somatomotor↔default mode network | -12.924 | 7.634 | -0.105 | 0.092 | 0.073 |
| Dorsal attention↔ventral attention network | 1.496 | 4.420 | 0.021 | 0.735 | 0.062 |
| Dorsal attention↔limbic network | 2.935 | 7.233 | 0.026 | 0.685 | 0.062 |
| Dorsal attention↔control network | 1.419 | 5.727 | 0.015 | 0.805 | 0.062 |
| Dorsal attention↔default mode network | -11.563 | 9.755 | -0.074 | 0.237 | 0.067 |
| Ventral attention↔limbic network | -9.701 | 7.073 | -0.085 | 0.172 | 0.069 |
| Ventral attention↔control network | 0.021 | 5.576 | 0.001 | 0.997 | 0.061 |
| Ventral attention↔default mode network | -3.115 | 7.847 | -0.025 | 0.692 | 0.062 |
| Limbic↔control network | -4.307 | 5.905 | -0.046 | 0.466 | 0.064 |
| Limbic↔default mode network | -9.132 | 6.462 | -0.088 | 0.159 | 0.069 |
| Control↔default mode network | -7.284 | 5.863 | -0.077 | 0.215 | 0.068 |
| The dependent variable was the Beck Depression Inventory II score at the first visit (t0), and the covariates were age, sex, and International Standard Classification of Education 1997 (ISCED97) level.  B, regression coefficient; β, standardized regression coefficient; R^2^, adjusted coefficient of determination; SE, standard error | | | | | |

**Supplementary Results S2: Detailed results from longitudinal analyses**

Results are from longitudinal repeated-measures ANCOVA with the respective FC_pos_ measures as the within-subject variables, sex as the between-subject factor, and age, educational level, time between scans, and ΔBDI-II (BDI-II_t1_ – BDI-II_t0_) as covariates. Here, we only report the results from the time x ΔBDI-II interaction.

We observed a significant time x ΔBDI-II interaction for intra-network FC_pos_ within the DAN (*p* = .040), for DMN segregation (*p* = .049), and for FC_pos_ between the SMN and the DMN (*p* = .037), but these results were no longer significant after Bonferroni correction. See Supplementary Tables S3 and S4 for detailed results.

| **Supplementary Table S3** Repeated-measures analyses of covariance for Δ Beck Depression Inventory II scores* versus longitudinal alterations of positive functional connectivity | | | | | |
| --- | --- | --- | --- | --- | --- |
| Within-subject variables (time) | *F* | df1 | df2 | *p* | η^2^_p_ |
| Visual network (intra) | 0.126 | 1 | 222 | 0.723 | 0.001 |
| Visual network (inter) | 0.017 | 1 | 222 | 0.896 | < 0.001 |
| Visual network (segregation) | 0.586 | 1 | 222 | 0.445 | 0.003 |
| Somatomotor network (intra) | 0.034 | 1 | 222 | 0.853 | < 0.001 |
| Somatomotor network (inter) | 0.006 | 1 | 222 | 0.939 | < 0.001 |
| Somatomotor network (segregation) | < 0.001 | 1 | 222 | 0.988 | < 0.001 |
| **Dorsal attention network (intra)** | **4.248** | **1** | **222** | **0.040** | **0.019** |
| Dorsal attention network (inter) | 0.095 | 1 | 222 | 0.758 | < 0.001 |
| Dorsal attention network (segregation) | 3.341 | 1 | 222 | 0.069 | 0.015 |
| Ventral attention network (intra) | 0.572 | 1 | 222 | 0.450 | 0.003 |
| Ventral attention network (inter) | 0.039 | 1 | 222 | 0.843 | < 0.001 |
| Ventral attention network (segregation) | 0.527 | 1 | 222 | 0.469 | 0.002 |
| Limbic network (intra) | 0.004 | 1 | 222 | 0.950 | < 0.001 |
| Limbic network (inter) | 0.361 | 1 | 222 | 0.549 | 0.002 |
| Limbic network (segregation) | 0.337 | 1 | 222 | 0.562 | 0.002 |
| Control network (intra) | 0.079 | 1 | 222 | 0.780 | < 0.001 |
| Control network (inter) | 1.561 | 1 | 222 | 0.213 | 0.007 |
| Control network (segregation) | 0.818 | 1 | 222 | 0.367 | 0.004 |
| Default mode network (intra) | 2.336 | 1 | 222 | 0.128 | 0.011 |
| Default mode network (inter) | 1.846 | 1 | 222 | 0.176 | 0.008 |
| **Default mode network (segregation)** | **3.928** | **1** | **222** | **0.049** | **0.018** |
| * Δ Beck Depression Inventory II scores was calculated as BDI-II_t1_ – BDI-II_t0_.  Covariates were age, sex, time between scans, and International Standard Classification of Education (ISCED97) level.  df1, degrees of freedom between groups; df2, degrees of freedom within groups; F, F-statistic; η^2^_p_, partial eta squared; inter, inter-network positive functional connectivity; intra, intra-network positive functional connectivity | | | | | |

| **Supplementary Table S4** Repeated-measures analyses of covariance for Δ Beck Depression Inventory II scores* versus longitudinal alterations of positive between-network functional connectivity | | | | | |
| --- | --- | --- | --- | --- | --- |
| Within-subject variables (time) | *F* | df1 | df2 | *p* | η^2^_p_ |
| Visual↔somatomotor network | 0.672 | 1 | 222 | 0.413 | 0.003 |
| Visual↔dorsal attention network | 1.027 | 1 | 222 | 0.312 | 0.005 |
| Visual↔ventral attention network | 0.968 | 1 | 222 | 0.326 | 0.004 |
| Visual↔limbic network | 0.088 | 1 | 222 | 0.767 | < 0.001 |
| Visual↔control network | 2.995 | 1 | 222 | 0.085 | 0.014 |
| Visual↔default mode network | 1.472 | 1 | 222 | 0.226 | 0.007 |
| Somatomotor↔dorsal attention network | 0.029 | 1 | 222 | 0.866 | < 0.001 |
| Somatomotor↔ventral attention network | 1.414 | 1 | 222 | 0.236 | 0.006 |
| Somatomotor↔limbic network | 0.451 | 1 | 222 | 0.502 | 0.002 |
| Somatomotor↔control network | 1.167 | 1 | 222 | 0.281 | 0.005 |
| **Somatomotor**↔**default mode network** | **4.386** | **1** | **222** | **0.037** | **0.020** |
| Dorsal attention↔ventral attention network | 0.275 | 1 | 222 | 0.601 | 0.001 |
| Dorsal attention↔limbic network | 0.430 | 1 | 222 | 0.513 | 0.002 |
| Dorsal attention↔control network | 0.166 | 1 | 222 | 0.684 | 0.001 |
| Dorsal attention↔default mode network | 0.003 | 1 | 222 | 0.959 | < 0.001 |
| Ventral attention↔limbic network | 1.815 | 1 | 222 | 0.179 | 0.008 |
| Ventral attention↔control network | 2.595 | 1 | 222 | 0.109 | 0.012 |
| Ventral attention↔default mode network | 1.159 | 1 | 222 | 0.283 | 0.005 |
| Limbic↔control network | 0.051 | 1 | 222 | 0.822 | < 0.001 |
| Limbic↔default mode network | 0.018 | 1 | 222 | 0.894 | < 0.001 |
| Control↔default mode network | 0.033 | 1 | 222 | 0.856 | < 0.001 |
| * Δ Beck Depression Inventory II score was calculated as BDI-II_t1_ – BDI-II_t0_.  Covariates were age, sex, time between scans, and International Standard Classification of Education (ISCED97) level.  df1, degrees of freedom between groups; df2, degrees of freedom within groups; F, F-statistic; η^2^_p_, partial eta squared | | | | | |

**Supplementary Results S3:** **Prediction of BDI-II alterations from baseline negative functional connectivity coefficients (FC­_neg_)**

Results from repeated-measures analyses of covariance (ANCOVA) with BDI-II at t0 and t1 as the within-subject variable, sex as the between-subject factor, and age, educational level, time between scans, and the respective intra-, inter-, or between-network negative functional connectivity (FC_neg_) measure or segregation score at t0 as covariates. Here, we report only the results for the time x FC_neg_ (t0) interaction effect (the results for the time x positive functional connectivity [FC_pos_] (t0) interaction effect can be found in the main text).

We found a significant positive BDI-II (time) x FC_neg_ (t0) interaction effect for the inter-network FC_neg_ of the default mode network (DMN; *F*(1, 241) = 6.864, *p* = .009, partial η^2^ = .028) and for the between-network FC_neg_ of the sensorimotor network (SMN) with the DMN (*F*(1, 241) = 5.773, *p* = .017, partial η^2^ = .024) and the dorsal network (DAN) with the DMN (*F*(1, 241) = 4.674, *p* = .032, partial η^2^ = .019). However, the effects were no longer significant after Bonferroni correction.

In addition, we found a trend-level effect for the inter-network FC_neg_ of the SMN (*p* = .077) and ventral attention network (VAN) segregation (*p* = .086) and for the between-network FC_negs_ of the visual network (VN) and the DMN (p = .071), the SMN and the control network (CN; p = .082), and the VAN and the CN (p = .083). See Supplementary Tables S5 and S6 for detailed results.

| **Supplementary Table S5** Repeated-measures analyses of covariance for baseline negative functional connectivity versus longitudinal changes in Beck Depression Inventory II scores | | | | | |
| --- | --- | --- | --- | --- | --- |
| Covariates* | *F* | df1 | df2 | *p* | η^2^_p_ |
| Visual network (intra) | 0.124 | 1 | 241 | 0.725 | 0.001 |
| Visual network (inter) | 0.542 | 1 | 241 | 0.462 | 0.002 |
| Visual network (segregation) | < 0.001 | 1 | 241 | 0.992 | < 0.001 |
| Somatomotor network (intra) | 0.255 | 1 | 241 | 0.614 | 0.001 |
| Somatomotor network (inter) | 3.158 | 1 | 241 | 0.077 | 0.013 |
| Somatomotor network (segregation) | 0.735 | 1 | 241 | 0.392 | 0.003 |
| Dorsal attention network (intra) | 0.907 | 1 | 241 | 0.342 | 0.004 |
| Dorsal attention network (inter) | 2.456 | 1 | 241 | 0.118 | 0.010 |
| Dorsal attention network (segregation) | 0.047 | 1 | 241 | 0.828 | < 0.001 |
| Ventral attention network (intra) | 1.163 | 1 | 241 | 0.282 | 0.005 |
| Ventral attention network (inter) | 1.322 | 1 | 241 | 0.251 | 0.006 |
| Ventral attention network (segregation) | 2.981 | 1 | 241 | 0.086 | 0.012 |
| Limbic network (intra) | 0.415 | 1 | 241 | 0.520 | 0.002 |
| Limbic network (inter) | 0.037 | 1 | 241 | 0.847 | < 0.001 |
| Limbic network (segregation) | 0.464 | 1 | 241 | 0.497 | 0.002 |
| Control network (intra) | 0.652 | 1 | 241 | 0.420 | 0.003 |
| Control network (inter) | 2.676 | 1 | 241 | 0.103 | 0.011 |
| Control network (segregation) | 1.441 | 1 | 241 | 0.231 | 0.006 |
| Default mode network (intra) | 0.004 | 1 | 241 | 0.947 | < 0.001 |
| **Default mode network (inter)** | **6.864** | **1** | **241** | **0.009** | **0.028** |
| Default mode network (segregation) | 2.708 | 1 | 241 | 0.101 | 0.011 |
| *Covariates were age, sex, time between scans, and International Standard Classification of Education 1997 (ISCED97) level.  df1, degrees of freedom between groups; df2, degrees of freedom within groups; F, F-statistic; η^2^_p_, partial eta squared; inter, inter-network negative functional connectivity; intra, intra-network negative functional connectivity | | | | | |

| **Supplementary Table S6** Repeated-measures analyses of covariance for baseline between-network negative functional connectivity versus longitudinal alterations of Beck Depression Inventory II scores | | | | | |
| --- | --- | --- | --- | --- | --- |
| Covariates* | *F* | df1 | df2 | *p* | η^2^_p_ |
| Visual↔somatomotor network | 1.303 | 1 | 241 | 0.255 | 0.005 |
| Visual↔dorsal attention network | 0.042 | 1 | 241 | 0.838 | < 0.001 |
| Visual↔ventral attention network | 0.210 | 1 | 241 | 0.647 | 0.001 |
| Visual↔limbic network | 0.242 | 1 | 241 | 0.623 | 0.001 |
| Visual↔control network | 1.183 | 1 | 241 | 0.278 | 0.005 |
| Visual↔default mode network | 3.297 | 1 | 241 | 0.071 | 0.014 |
| Somatomotor↔dorsal attention network | 0.114 | 1 | 241 | 0.736 | < 0.001 |
| Somatomotor↔ventral attention network | 0.111 | 1 | 241 | 0.739 | < 0.001 |
| Somatomotor↔limbic network | 0.075 | 1 | 241 | 0.785 | < 0.001 |
| Somatomotor↔control network | 3.049 | 1 | 241 | 0.082 | 0.013 |
| **Somatomotor**↔**default mode network** | **5.773** | **1** | **241** | **0.017** | **0.024** |
| Dorsal attention↔ventral attention network | < 0.001 | 1 | 241 | 0.995 | < 0.001 |
| Dorsal attention↔limbic network | 0.272 | 1 | 241 | 0.602 | 0.001 |
| Dorsal attention↔control network | 2.035 | 1 | 241 | 0.155 | 0.009 |
| **Dorsal attention**↔**default mode network** | **4.674** | **1** | **241** | **0.032** | **0.019** |
| Ventral attention↔limbic network | 2.068 | 1 | 241 | 0.152 | 0.009 |
| Ventral attention↔control network | 3.031 | 1 | 241 | 0.083 | 0.013 |
| Ventral attention↔default mode network | 2.556 | 1 | 241 | 0.111 | 0.011 |
| Limbic↔control network | 1.195 | 1 | 241 | 0.275 | 0.005 |
| Limbic↔default mode network | 0.022 | 1 | 241 | 0.881 | < 0.001 |
| Control↔default mode network | 0.162 | 1 | 241 | 0.687 | 0.001 |
| *Covariates were age, sex, time between scans, and International Standard Classification of Education 1997 (ISCED97) level.  df1, degrees of freedom between groups; df2, degrees of freedom within groups; F, F-statistic; η^2^_p_, partial eta squared | | | | | |

**Supplementary Results S4: Detailed results of the associations between baseline FC and longitudinal changes in BDI-II scores**

Results are from repeated measures ANCOVA with the t0 and t1 BDI-II values as the within-subject variable, sex as the between-subject factor, and age, educational level, time between scans, and the respective intra-, inter- or between-network FC_pos_ measure or segregation score at t0 as covariates. Here, we only report results from the time x FC_pos_ (t0) interaction effect.

Analyses revealed a significant association of longitudinal changes in BDI-II with baseline intra-network FC_pos_ (*p* = .004) and segregation of the VAN (*p* = .043) and with baseline intra-network FC_pos_ (*p* = .010) and segregation of the DMN (*p* = .040). However, only the association with baseline intra-network FC_pos_ and segregation of the VAN remained significant after applying the Bonferroni correction. See Supplementary Tables S7 and S8 for detailed results.

| **Supplementary Table S7** Repeated-measures analyses of covariance for baseline FC_pos_ versus longitudinal changes in Beck Depression Inventory II scores | | | | | |
| --- | --- | --- | --- | --- | --- |
| Covariate* | *F* | df1 | df2 | *p* | η^2^_p_ |
| Visual network (intra) | 0.293 | 1 | 241 | 0.589 | 0.001 |
| Visual network (inter) | 2.030 | 1 | 241 | 0.155 | 0.008 |
| Visual network (segregation) | 0.202 | 1 | 241 | 0.654 | 0.001 |
| Somatomotor network (intra) | 1.132 | 1 | 241 | 0.289 | 0.005 |
| Somatomotor network (inter) | 2.307 | 1 | 241 | 0.130 | 0.010 |
| Somatomotor network (segregation) | 0.042 | 1 | 241 | 0.838 | < 0.001 |
| Dorsal attention network (intra) | 0.199 | 1 | 241 | 0.656 | 0.001 |
| Dorsal attention network (inter) | 2.457 | 1 | 241 | 0.118 | 0.010 |
| Dorsal attention network (segregation) | 0.513 | 1 | 241 | 0.474 | 0.002 |
| **Ventral attention network (intra)** | **8.560** | **1** | **241** | **0.004** | **0.035** |
| Ventral attention network (inter) | 2.100 | 1 | 241 | 0.149 | 0.009 |
| **Ventral attention network (segregation)** | **4.141** | **1** | **241** | **0.043** | **0.017** |
| Limbic network (intra) | 0.978 | 1 | 241 | 0.324 | 0.004 |
| Limbic network (inter) | 0.121 | 1 | 241 | 0.728 | 0.001 |
| Limbic network (segregation) | 0.695 | 1 | 241 | 0.405 | 0.003 |
| Control network (intra) | 0.511 | 1 | 241 | 0.476 | 0.002 |
| Control network (inter) | 0.014 | 1 | 241 | 0.907 | < 0.001 |
| Control network (segregation) | 0.377 | 1 | 241 | 0.540 | 0.002 |
| **Default mode network (intra)** | **6.725** | **1** | **241** | **0.010** | **0.028** |
| Default mode network (inter) | 0.107 | 1 | 241 | 0.744 | < 0.001 |
| **Default mode network (segregation)** | **4.255** | **1** | **241** | **0.040** | **0.018** |
| *Covariates were age, sex, time between scans, and International Standard Classification of Education (ISCED97) level.  df1, degrees of freedom between groups; df2, degrees of freedom within groups; F, F-statistic; inter, inter-network positive functional connectivity; intra, intra-network positive functional connectivity; η^2^_p_, partial eta squared | | | | | |

| **Supplementary Table S8** Repeated-measures analyses of covariance for baseline between-network FC_pos_ versus longitudinal changes in Beck Depression Inventory II scores | | | | | |
| --- | --- | --- | --- | --- | --- |
| Covariates* | *F* | df1 | df2 | *p* | η^2^_p_ |
| Visual↔somatomotor network | 2.982 | 1 | 241 | 0.086 | 0.012 |
| Visual↔dorsal attention network | 1.360 | 1 | 241 | 0.245 | 0.006 |
| Visual↔ventral attention network | 1.574 | 1 | 241 | 0.211 | 0.007 |
| Visual↔limbic network | 0.044 | 1 | 241 | 0.833 | < 0.001 |
| Visual↔control network | 0.593 | 1 | 241 | 0.442 | 0.002 |
| Visual↔default mode network | 1.012 | 1 | 241 | 0.316 | 0.004 |
| Somatomotor↔dorsal attention network | 2.541 | 1 | 241 | 0.112 | 0.011 |
| Somatomotor↔ventral attention network | 3.226 | 1 | 241 | 0.074 | 0.013 |
| Somatomotor↔limbic network | 0.342 | 1 | 241 | 0.559 | 0.001 |
| Somatomotor↔control network | 1.565 | 1 | 241 | 0.212 | 0.007 |
| Somatomotor↔default mode network | 2.225 | 1 | 241 | 0.137 | 0.009 |
| Dorsal attention↔ventral attention network | 1.034 | 1 | 241 | 0.308 | 0.004 |
| Dorsal attention↔limbic network | 0.475 | 1 | 241 | 0.491 | 0.002 |
| Dorsal attention↔control network | 0.260 | 1 | 241 | 0.610 | 0.001 |
| Dorsal attention↔default mode network | 0.077 | 1 | 241 | 0.781 | < 0.001 |
| Ventral attention↔limbic network | 0.514 | 1 | 241 | 0.474 | 0.002 |
| Ventral attention↔control network | 0.171 | 1 | 241 | 0.680 | 0.001 |
| Ventral attention↔default mode network | 0.146 | 1 | 241 | 0.703 | 0.001 |
| Limbic↔control network | 0.252 | 1 | 241 | 0.616 | 0.001 |
| Limbic↔default mode network | 1.734 | 1 | 241 | 0.189 | 0.007 |
| Control↔default mode network | 2.118 | 1 | 241 | 0.147 | 0.009 |
| *Covariates were age, sex, time between scans, and International Standard Classification of Education (ISCED97) level.  df1, degrees of freedom between groups; df2, degrees of freedom within groups; F, F-statistic; η^2^_p,_ partial eta squared | | | | | |

**Supplementary Results S5:** **Detailed analysis of the relationship between VAN regions of interest and changes in BDI-II scores**

We used the seven-network Schaefer 400 parcellation framework (https://github.com/ThomasYeoLab/CBIG/tree/master/stable_projects/brain_parcellation/Schaefer2018_LocalGlobal/Parcellations/MNI) to calculate positive FC for each region of interest within the VAN. Subsequently, we examined the relationship between changes in BDI-II scores and hemispheric FC of the VAN in the right and left hemispheres and assessed the association between BDI-II changes and FC of individual VAN regions of interest. Detailed results are shown in Supplementary Tables S9 and S10.

| **Supplementary Table S9** Repeated-measures analyses of covariance for baseline positive functional connectivity within left hemispheric ventral attention network regions of interest versus longitudinal changes in Beck Depression Inventory II scores | | | | | | |
| --- | --- | --- | --- | --- | --- | --- |
| Covariates* | *F* | df1 | df2 | *p* | *p*-FDR | η^2^_p_ |
| Parietal operculum 1 | 3.908 | 1 | 237 | 0.049 | 0.135 | 0.016 |
| Parietal operculum 2 | 0.007 | 1 | 237 | 0.935 | 0.935 | < 0.001 |
| Parietal operculum 3 | 3.156 | 1 | 237 | 0.077 | 0.154 | 0.013 |
| Parietal operculum 4 | 0.131 | 1 | 237 | 0.717 | 0.789 | 0.001 |
| Temporal-occipital region 1 | 0.052 | 1 | 237 | 0.820 | 0.859 | < 0.001 |
| Frontal operculum insula 1 | 0.920 | 1 | 237 | 0.338 | 0.438 | 0.004 |
| Frontal operculum insula 2 | 4.172 | 1 | 237 | 0.042 | 0.133 | 0.017 |
| Frontal operculum insula 3 | 1.706 | 1 | 237 | 0.193 | 0.303 | 0.007 |
| Frontal operculum insula 4 | 2.785 | 1 | 237 | 0.096 | 0.177 | 0.012 |
| Frontal operculum insula 5 | 1.061 | 1 | 237 | 0.304 | 0.418 | 0.004 |
| Frontal operculum insula 6 | 3.708 | 1 | 237 | 0.055 | 0.135 | 0.015 |
| Frontal operculum insula 7 | 4.814 | 1 | 237 | 0.029 | 0.107 | 0.020 |
| **Frontal operculum insula 8** | **10.846** | **1** | **237** | **0.001** | **0.025** | **0.044** |
| Frontal operculum insula 9 | 5.959 | 1 | 237 | 0.015 | 0.068 | 0.025 |
| Lateral prefrontal cortex 1 | 1.473 | 1 | 237 | 0.226 | 0.331 | 0.006 |
| Medial 1 | 0.522 | 1 | 237 | 0.471 | 0.575 | 0.002 |
| Medial 2 | 3.556 | 1 | 237 | 0.061 | 0.133 | 0.015 |
| Medial 3 | 0.220 | 1 | 237 | 0.639 | 0.740 | 0.001 |
| Medial 4 | 6.735 | 1 | 237 | 0.010 | 0.055 | 0.028 |
| Medial 5 | 2.104 | 1 | 237 | 0.148 | 0.251 | 0.009 |
| Medial 6 | 8.177 | 1 | 237 | 0.005 | 0.051 | 0.033 |
| Medial 7 | 6.948 | 1 | 237 | 0.009 | 0.066 | 0.028 |
| *Covariates were age, sex, time between scans, and International Standard Classification of Education (ISCED97) level.  df1, degrees of freedom between groups; df2, degrees of freedom within groups; F, F-statistic; FDR, false discovery rate; η^2^_p_, partial eta squared | | | | | | |

| **Supplementary Table S10** Repeated-measures analyses of covariance for baseline FC_pos_ within the right hemispheric ventral attention network regions of interest versus longitudinal changes in Beck Depression Inventory II scores | | | | | | |
| --- | --- | --- | --- | --- | --- | --- |
| Covariates* | *F* | df1 | df2 | *p* | *p*-FDR | η^2^_p_ |
| Temporal-occipital-parietal region 1 | 1.245 | 1 | 237 | 0.264 | 0.287 | 0.005 |
| **Temporal-occipital-parietal region 2** | **6.337** | **1** | **237** | **0.012** | **0.031** | **0.026** |
| Temporal-occipital-parietal region 3 | 2.606 | 1 | 237 | 0.108 | 0.142 | 0.011 |
| **Temporal-occipital-parietal region 4** | **7.310** | **1** | **237** | **0.007** | **0.031** | **0.030** |
| **Temporal-occipital-parietal region 5** | **8.120** | **1** | **237** | **0.005** | **0.030** | **0.033** |
| **Temporal-occipital-parietal region 6** | **6.192** | **1** | **237** | **0.014** | **0.031** | **0.025** |
| Temporal-occipital-parietal region 7 | 2.301 | 1 | 237 | 0.131 | 0.156 | 0.010 |
| Precentral cortex 1 | 3.518 | 1 | 237 | 0.062 | 0.097 | 0.015 |
| Frontal operculum insula 1 | 2.152 | 1 | 237 | 0.144 | 0.163 | 0.009 |
| **Frontal operculum insula 2** | **6.855** | **1** | **237** | **0.009** | **0.029** | **0.028** |
| Frontal operculum insula 3 | 4.726 | 1 | 237 | 0.031 | 0.059 | 0.020 |
| Frontal operculum insula 4 | 3.418 | 1 | 237 | 0.066 | 0.097 | 0.014 |
| **Frontal operculum insula 5** | **5.299** | **1** | **237** | **0.022** | **0.046** | **0.022** |
| Frontal operculum insula 6 | 2.319 | 1 | 237 | 0.129 | 0.161 | 0.010 |
| **Frontal operculum insula 7** | **6.465** | **1** | **237** | **0.012** | **0.032** | **0.027** |
| **Frontal operculum insula 8** | **8.736** | **1** | **237** | **0.003** | **0.029** | **0.036** |
| Lateral prefrontal cortex 1 | 0.823 | 1 | 237 | 0.365 | 0.381 | 0.003 |
| Medial 1 | 2.673 | 1 | 237 | 0.103 | 0.144 | 0.011 |
| **Medial 2** | **6.997** | **1** | **237** | **0.009** | **0.031** | **0.029** |
| Medial 3 | 3.838 | 1 | 237 | 0.051 | 0.085 | 0.016 |
| **Medial 4** | **8.031** | **1** | **237** | **0.005** | **0.025** | **0.033** |
| **Medial 5** | **9.011** | **1** | **237** | **0.003** | **0.037** | **0.037** |
| Medial 6 | 4.559 | 1 | 237 | 0.034 | 0.060 | 0.019 |
| **Medial 7** | **13.082** | **1** | **237** | **< 0.001** | **0.009** | **0.052** |
| Medial 8 | 0.723 | 1 | 237 | 0.396 | 0.396 | 0.003 |
| *Covariates were age, sex, time between scans, and International Standard Classification of Education (ISCED97) level.  df1, degrees of freedom between groups; df2, degrees of freedom within groups; F, F-statistic; FDR, false discovery rate; η^2^_p_, partial eta squared | | | | | | |
